# Co_3_O_4_-NP embedded mesoporous carbon rod with enhanced electrocatalytic conversion in lithium-sulfur battery

**DOI:** 10.1038/s41598-018-34195-z

**Published:** 2018-10-31

**Authors:** Shaofeng Wang, Xianhua Hou, Zeming Zhong, Kaixiang Shen, Guangzu Zhang, Lingmin Yao, Fuming Chen

**Affiliations:** 1 Guangdong Engineering Technology Research Center of Efficient Green Energy and Environment Protection Materials, Guangdong Provincial Key Laboratory of Quantum Engineering and Quantum Materials, School of Physics and Telecommunication Engineering, South China Normal University, Guangzhou, 510006 PR China; 2 School of Optical and Electronic Information, Huazhong University of Science and Technology, Wuhan, 430074 PR China; 3 School of Physics and Electronic Engineering, Guangzhou University, Guangzhou, 510006 PR China

## Abstract

Lithium-sulfur battery has been considered to be one of the promising alternatives to the traditional lithium-ion battery due to its high theoretical energy density and saving-cost. However, the sluggish reaction during the decomposition of lithium sulfide results in a low specific capacity and poor cycling stability. Herein Co_3_O_4_ nano-particle embedded mesoporous carbon rod (Co_3_O_4_@MCR) was prepared through a template method to accommodate sulfur as cathode of lithium-sulfur battery. The resultant composite was characterized by Raman spectra, XRD, TEM, SEM, etc. The electrochemical investigation demonstrated that Co_3_O_4_@MCR composite exhibits enhanced electrocatalytic performance in lithium-sulfur battery, which was confirmed by cyclic voltammograms, galvanostatic charge-discharge testing, and study of sulfide oxidation using linear sweep voltammetry. With the current density of 0.2 A/g, the specific discharge capacity can be achieved up to more than 1000 mAh/g after 100 cycles. The enhanced electrocatalytic conversion from Co_3_O_4_@MCR leads to a low over-potential, fast lithium-ion kinetics and sulfide oxidation reaction.

## Introduction

How to meet the rapidly growing demand of energy storage for electric vehicles and smart devices is a prominent issue. Traditional lithium-ion battery still serves as one of the most important commercial power storage devices^1^. However, lithium-ion battery is facing great challenges to meet the rapidly growing demand of energy storage^2,3^, and new commercial battery system with higher energy density and longer service life is required^4^. Lithium-sulfur battery (LSB) system is supposed to possess some premier features such as high theoretical energy density of ~2600 Wh kg^−1^ ^5,6^, low cost and natural abundance of the active material (sulfur)^7–10^. It has been paid much attention over the past decade^11–15^, and is considered as the best practical alternative to traditional lithium ion battery. In lithium-sulfur battery system, sulfur works as cathode active material which provides an extremely high reversible specific capacity of ~1600 mAh/g with the formation of lithium sulfide (Li_2_S)^16–18^. Despite the impressive theoretical specific capacity, in actual application sulfur cathode suffers from several key drawbacks. For example, the volume expanding during discharge process can reaches as large as ~80%^19^, which may destroy the cathode construction, causing the shedding of the active sulfur. The infamous lithium polysulfides in electrolyte can erode the active material and fade the cycling performance of LSBs^20,21^. To settle down these problems, researches are focused on constructing functional cathodes, increasing lithium salt concentration in electrolytes, and introducing additives in electrolytes^22–25^.

Another issue is over-potential problem during the charge-discharge process, which is directly associated with energy efficiency of battery. Over-potential is determined by the difference between consumed energy during charge and released energy during discharge. Over-potential problem has been investigated^26–28^. In the previous reports some sulfides are able to reduce the over-potential because the energy barrier for the decomposition of Li_2_S is associated with the binding between isolated Li-ions and the sulfur from sulfides^29^. Co_3_O_4_-based composites are reported to show excellent catalytic performances in many fields including oxygen reduction reaction^30–32^, lithium-air battery^33–35^, and catalytic hydrogenation^36,37^, etc. In lithium-sulfur battery, cobalt composite^38–41^ demonstrated the catalytic function.

In this work, a facile large-scale method is presented to prepare Co_3_O_4_-NP embedded mesoporous carbon rod (Co_3_O_4_@MCR) through an SBA-15 silica template method followed with an impregnation process, and the composite is used as an efficient carrier for accommodating sulfur as cathode with high catalytic performance for lithium-sulfur battery. Compared with the bare mesoporous carbon rod (MCR) prepared from SBA-15 template, Co_3_O_4_@MCR composite demonstrates enhanced electrocatalytic performance when applied as cathode with the deposition of sulfur in lithium-sulfur battery. Specially, an obviously low over-potential can be observed in S-Co_3_O_4_@MCR cathode during charge/discharge processes. In addition, the testing of sulfide oxidation reaction was conducted using linear sweep voltammetry method, which is as auxiliary technique to evaluate the catalytic performance of the Co_3_O_4_@MCR composite. Electrochemical performances were demonstrated for the as-prepared samples, and possible reasons (carbon construction and catalytic activity are enhanced by introducing Co_3_O_4_) for such favorable performances were discussed.

## Experimental

### Synthesis of the Co_3_O_4_@MCR composite

Co_3_O_4_-NP mesoporous carbon rod was prepared using silica SBA-15 as template. In typical process, 1.75 mmol sucrose was dissolved into 2.5 mL deionized water to form a homogenous solution. Then 1.6 mmol H_3_BO_4_ and 44.0 μL concentrated H_2_SO_4_ (98%) was added into the solution. After 0.5 g SBA-15 was added into the solution, the as-received mixture was treated in 100 °C for 6 h and 160 °C for 6 h. Then the heated mixture was treated in 900 °C for 3 h under Ar atmosphere for carbonization and the received powder was impregnated by ethanol solution which contains 0.5 mmol of Co(NO_3_)_2_. After the ethanol was evaporated completely, the mixture was treated under 300 °C for 3 h in air. The removal of SBA-15 was taken using 2M NaOH solution. After rinsed and dried, the Co_3_O_4_@MCR composite was received. For mesoporous carbon rod, the synthesis processes are similar without the impregnating process.

### Characterization

X-ray diffraction patterns (XRD) for the composites were conducted using Panalytical Xpert-pro (Cu K-α radiation, λ = 1.5406 Å). Raman spectra were characterized by a RENISHAW inVia Raman Microscope. A BELSORP II instrument was used to obtain the nitrogen adsorption-desorption isotherms. Transmission electron microscopy (TEM) was carried out by JEM-2100HR. Surficial morphologies were detected by field emission scanning electron microscopy (FE-SEM) using ZEISS ULTRA 55 microscopy and energy dispersive X-ray (EDX) elemental mapping information of the as-prepared sample was collected using Tescan Mira3 field emission scanning electron microscope. UV-vis adsorption spectra of the reacted electrolyte were collected using Shimadzu-2550 UV-visible spectrophotometer in linear sweep voltammetry condition.

### Electrochemical measurement

In S-Co_3_O_4_@MCR composite, sulfur to Co_3_O_4_@MCR mass ratio is controlled at 7:3. S-Co_3_O_4_@MCR, Super P and LA-132 were mixed at the weight ratio of 70:15:15 using a planetary mixer in deionized water. The homogenous slurry was coated on aluminium foil and dried in vacuum oven at 60 °C for 12 hours. The film was then punched into circular shape with diameter of 12 mm. Each cathode contains approximate 1.5 mg of S. The electrochemical measurements were conducted using CR2432 coin type cells, consisting of S-Co_3_O_4_@MCR cathode, electrolyte (electrolyte/S ratio is ~7 mL/g), separator and metal lithium anode. The electrolyte was prepared with 1 M bis(trifluoromethane)sulfonimide lithium in a mixed solvent of 1,3-dioxolane and 1,2-dimethoxyethane (v/v = 1:1), with 1 wt% LiNO_3_. Solartron 1400 Celltest System was used for cyclic voltammetry and galvanostatic test in the coin cell. Voltage range of cyclic voltammograms were controlled at the range of 1.5 and 3.0 V with a scan rate of 0.05 mV s^−1^. Galvanostatic test was performed at the voltage window of 1.7 to 2.8 V. Linear sweep voltammetry (LSV) was measured using a CHI electrochemical workstation with a standard three-electrode configuration in 0.1 M Li_2_S/methanol solution. Glassy carbon electrode with diameter of 4 mm was used as the working electrode, saturated Ag/AgCl electrode as reference, and platinum sheet as counter electrode. Herein the working electrode was prepared by the following steps: 60.0 mg of Co_3_O_4_@MCR, 20.0 mg of polyvinylidene fluoride and 20.0 mg of super P were dispersed in 0.5 mL of N-methyl pyrrolidone by mild sonication for 2 h to form a homogeneous ink. Then, a 10 μL aliquot of as-prepared ink was transferred onto the surface of the GC substrate. The catalyst layer on the GC electrode was dried under an infrared lamp before LSV measurements. The LSV measurements were conducted at the voltage range of −0.7 V to −0.1 V at a scan rate of 5 mV s^−1^.

## Results and Discussion

**Figure 1:**
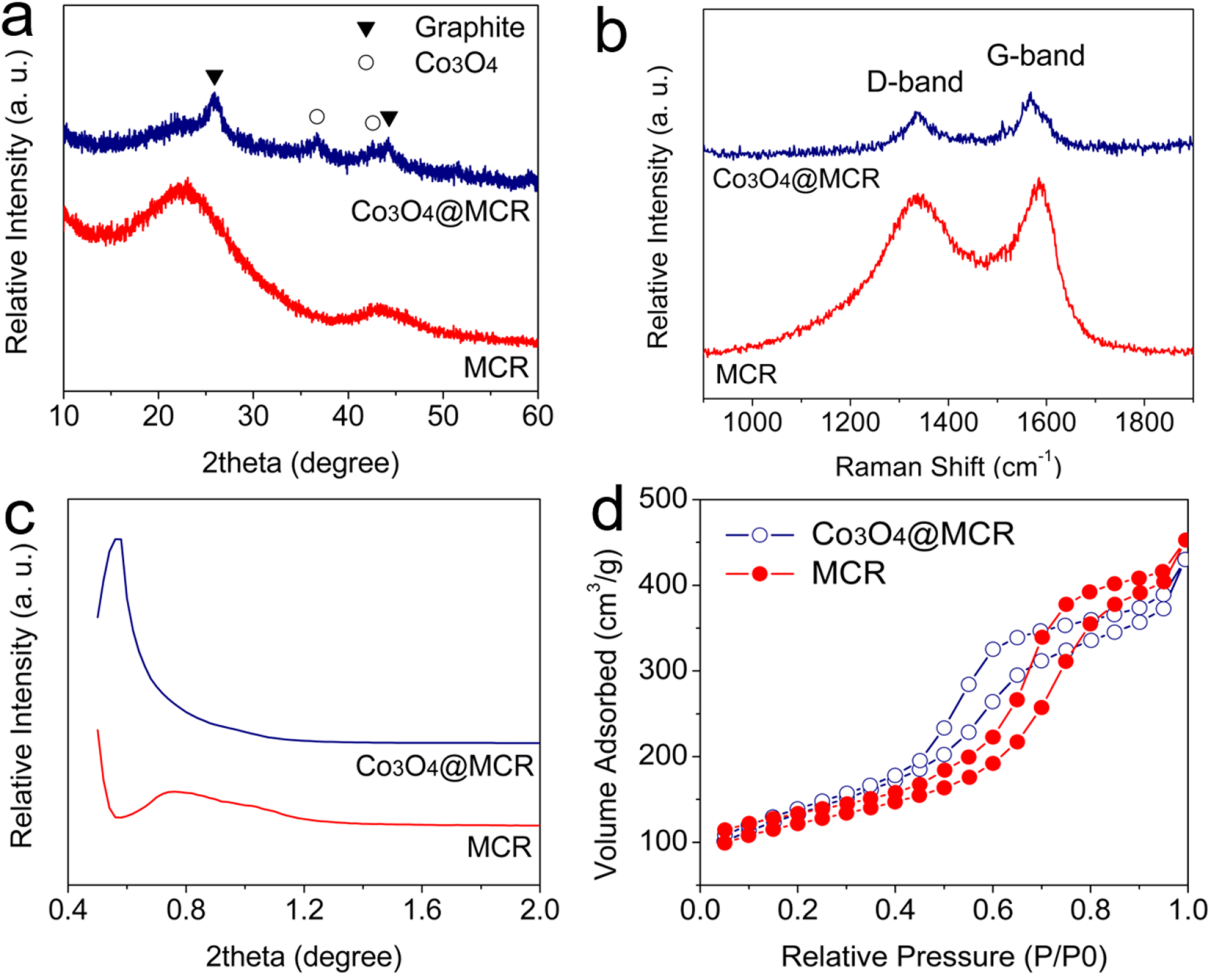
(**a**) X-ray diffraction patterns, (**b**) Raman spectra, (**c**) small angle X-ray diffraction patterns and (**d**) N_2_ adsorption/desorption isotherms of the as-prepared Co_3_O_4_@MCR and MCR samples.

Figure 1(a) demonstrates the X-ray diffraction scanning results of MCR and Co_3_O_4_@MCR. For MCR sample, no prominent peak is observed except for two broad bumps located at ~22.0° and 43.0° which are related to amorphous carbon. For Co_3_O_4_@MCR, the peaks located at 26.0° and 44.3° can be indexed to graphite crystal and peaks at 36.8° and 42.8° were assigned to spinal Co_3_O_4_, indicating the composite of graphite and Co_3_O_4_. In addition, the impregnation progress not only results in the deposition of spinel Co_3_O_4_, but also causes crystalline formation of amorphous carbon. Compared with amorphous carbon, graphite possesses high electronic conductivity and robust construction, which can enhance the electrochemical performance of lithium-sulfur battery. To investigate the carbon crystalline in both MCR and Co_3_O_4_@MCR samples, Raman spectra were carried out, as shown in Fig. 1(b). The band at 1335 cm^−1^ is referred to D-band from lattice breaking on the sp2-hybridized carbons, and the band at 1575 cm^−1^ from G-band reflecting the crystal planes of the sp2-hybridized carbon atoms. The intensity ratio of the D-band to G-band (I_D_ to I_G_ ratio) indicates the distortion of carbon during the pyrolysis under elevated temperature. I_D_/I_G_ ratio in MCR is calculated to be 1.00 while the ratio in Co_3_O_4_@MCR is 0.68, implying the relative better crystallinity in Co_3_O_4_@MCR due to the integrated sp2-hybridized carbon atoms.

To accommodate the active sulfur material and increase the conductivity, it is necessary to create some sufficient pores at supporting carbon substrates or framework materials. The mesoporous substrate can improve the electrode reaction stability. Herein, mesoporous SBA-15 was used as template to prepare Co_3_O_4_@MCR and MCR. As known, SBA-15 silica contains large amount of ordered mesopores^42,43^. The low-angle X-ray diffraction patterns of the as-prepared Co_3_O_4_@MCR composite were measured to obtain basic information about the mesoporous structure. For comparison, bare amorphous MCR sample without Co_3_O_4_ catalyst was also measured. As shown in Fig. 1(c), both Co_3_O_4_@MCR and the amorphous carbon MCR showed prominent diffraction peaks. The ordered mesoporous structures inherited from SBA-15 were still detected in the two samples. It is noted that the prominent peak is located at 0.57° in Co_3_O_4_@MCR at low-angle diffraction patterns, however, it blue-shifts to less than 0.50° in MCR sample, implying that the pore size is slightly smaller in Co_3_O_4_@MCR than that of MCR due to the shrink of the carbon catalyzed by cobalt element. In addition, the diffraction intensity of the prominent peak is lower in Co_3_O_4_@MCR sample, indicating that the embedment of Co_3_O_4_ disturbs the ordered arrangement of the mesopores during the pore formation. It is well known that transition metal oxides are good catalyst for pyrolysis reaction of organics^44,45^, which changes the pyrolysis behavior of the organic carbon precursor, and thus, affects the pore distribution inside the sample of Co_3_O_4_@MCR.

Pore size and distribution of Co_3_O_4_@MCR and MCR was further investigated by N_2_ adsorption/desorption isotherms, as demonstrated in Fig. 1(d). Both samples showed typical IV nitrogen adsorption/desorption isotherms with hysteresis loops, indicating the presence of mesopores. The specific surface area is calculated as 475.3 m^2^/g for Co_3_O_4_@MCR, and 433.7 m^2^/g for MCR based on Brunauer-Emmett-Teller method. XRD and Raman spectra result exhibited that the Co_3_O_4_ embedment disturbs the crystalline structure and the pore order arrangement in MCR, but the similar specific surface area imply that the embedment of cobalt element neither shrinks nor expands the specific surface area of the carbon substrate. The pore size distribution result based on Barret-Joyner-Halenda method is shown in Fig. S1. The pore size of Co_3_O_4_@MCR is distributed around 4 nm while MCR is around 7 nm. This result is consistent with the low-angle X-ray diffraction, further indicating the graphitization influence of Co_3_O_4_ on the carbon substrate.

**Figure 2:**
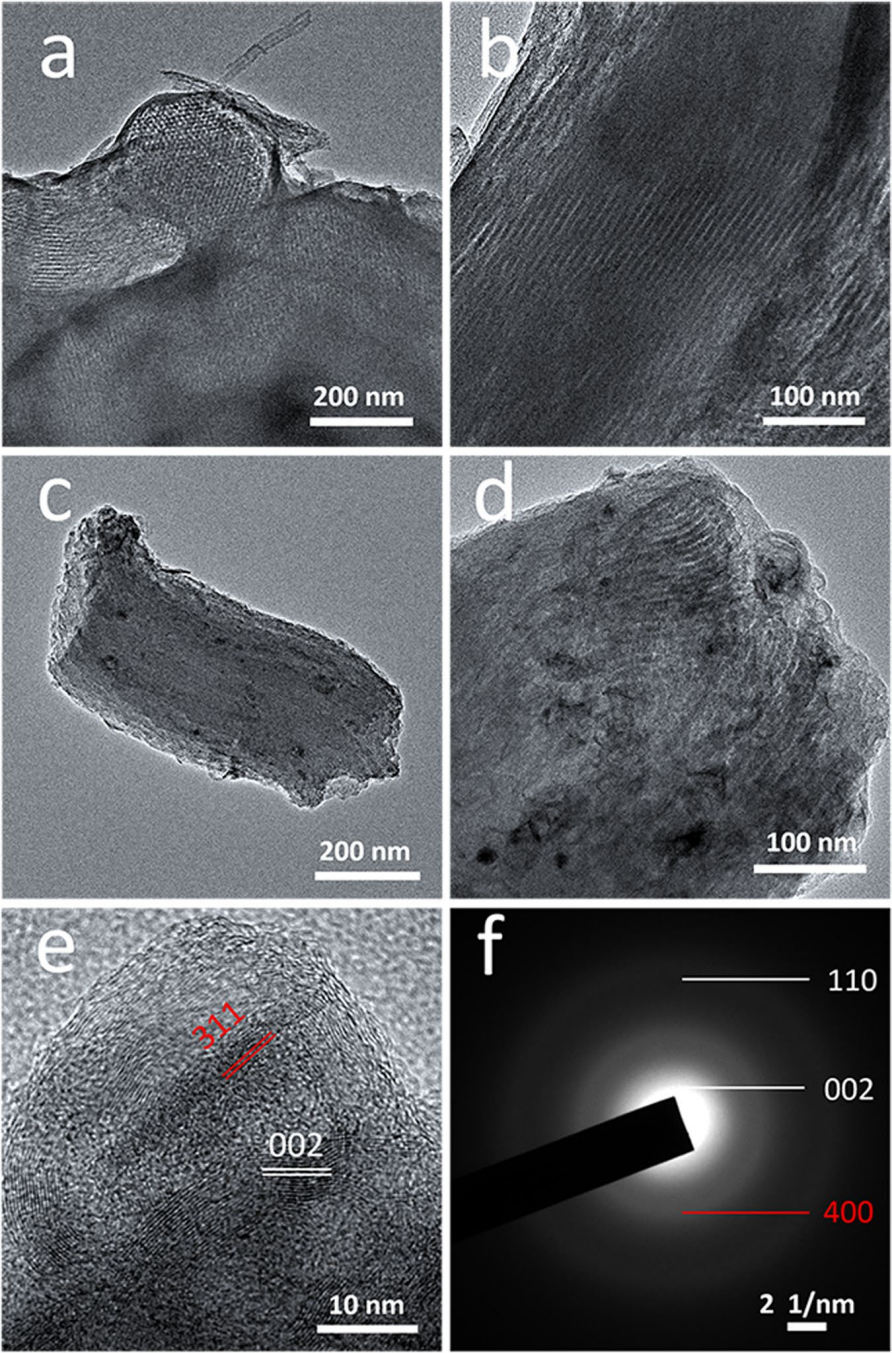
(**a**) Low magnification and (**b**) high magnification TEM images of the as-prepared MCR sample; (**c**) Low magnification, (**d**) high magnification and (**e**) HR-TEM images of the as-prepared Co_3_O_4_@MCR sample; (**f**) selected area electron diffraction patterns of the as-prepared Co_3_O_4_@MCR sample.

TEM was conducted to insight the inner nanostructure of the samples and understand the distribution of Co_3_O_4_ in the carbon substrate. Figure 2(a,b) are the TEM images of the MCR composite in low and high magnifications, respectively. The ordered pore channels are clearly observed, and the diameter of the pore is around 7.6 nm, which is in accordance with the low-angle X-ray diffraction and BJH pore distribution results. Figure 2(c,d) are the TEM images of Co_3_O_4_@MCR in low and high magnifications; and some scattered dark contrasts of the image were clearly observed. From the high magnification image, the dark contrasts are conveyed to be in the size varied from 10 to 40 nm imbedding in the substrate. HR-TEM image for Co_3_O_4_@MCR was measured to offer evidence for the phase construction of the dark contrasts and the substrate with the typical picture shown in Fig. 2(e). The substrate shows visible diffraction patterns with distances of 0.33 nm (marked with white lines in Fig. 2(e)), which could be related to the plane of (002) for crystal graphite, and this result can be reasonably assigned to the catalysis behavior of cobalt for carbon substrate. And the fringe patterns for the contrast zones were observed with distances of 0.25 nm (marked with red lines in Fig. 2(e)), indexed well with the diffraction planes of (311) for cubic Co_3_O_4_. It confirms that nanosized Co_3_O_4_ scattered in the carbon substrate. Furthermore, selected area electron diffraction (SAED) patterns in Fig. 2(f) provide the direct supporting evidence to the co-existence of Co_3_O_4_ and graphite phases. As the distinct ring patterns show, the ring patterns marked as (002) and (110) in white refer to graphite and the ring pattern marked as (400) in red refer to cubic Co_3_O_4_.

**Figure 3:**
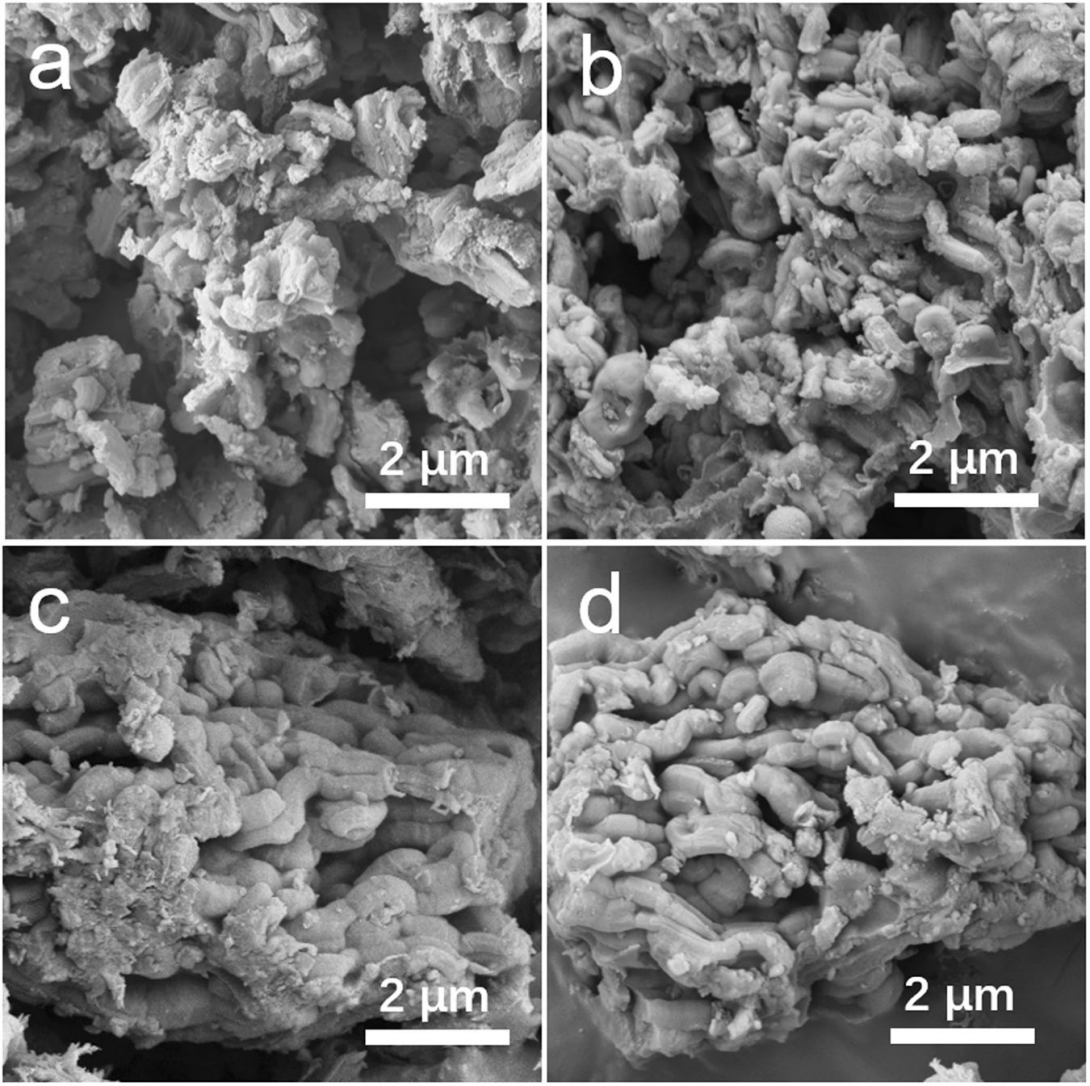
FE-SEM images of the as-prepared (**a**) MCR, (**b**) S-MCR, (**c**) Co_3_O_4_@MCR and (**d**) S-Co_3_O_4_@MCR samples.

Figure 3 demonstrated the low magnification FE-SEM images of the as-prepared samples. Figure 3(a,c) are images for the exterior morphology of MCR and Co_3_O_4_@MCR samples, respectively. Both the samples show similar block morphology constructed by small stick like particle which has diameter of about 500 nm and length of about 1 μm. As investigated, both the MCR and Co_3_O_4_@MCR composites have inner pore structure and relatively high specific surface area, however, the capability for sulfur accommodation is not clear. For this case, low magnification SEM images of both S-MCR and S-Co_3_O_4_@MCR composites were conducted and the results were shown in Fig. 3(b,d). Low magnification images are applied for the purpose to provide persuasive result and reduce the occasional error version on the composites. According to these images, there is bear aggregation on the surfaces or surround the body of MCR and Co_3_O_4_@MCR composites, indicating the excellent accommodation of sulfur in both MCR and Co_3_O_4_@MCR composites.

In addition, the EDX elemental mapping for S-Co_3_O_4_@MCR sample was carried out to confirm the accommodation of sulfur element in the composite as shown in Fig. S2. The image of carbon elemental mapping is distributed through the whole sample, and the weak mapping signal from Co indicating that Co_3_O_4_ may be embedded in the carbon matrix. And as expected, the sulfur mapping signal is spreading though the whole sample district, shown that sulfur is homogeneously distributed in the composite. The weight ratio of S in S-Co_3_O_4_@MCR composite was investigated using thermogravimetric analysis (TGA) as the result demonstrated in Fig. S3. According to the result, the weight ratio of S in S-Co_3_O_4_@MCR composite is 57.1%. Note that, the weight ratio of Co_3_O_4_ in Co_3_O_4_@MCR is 24.2%.

**Figure 4:**
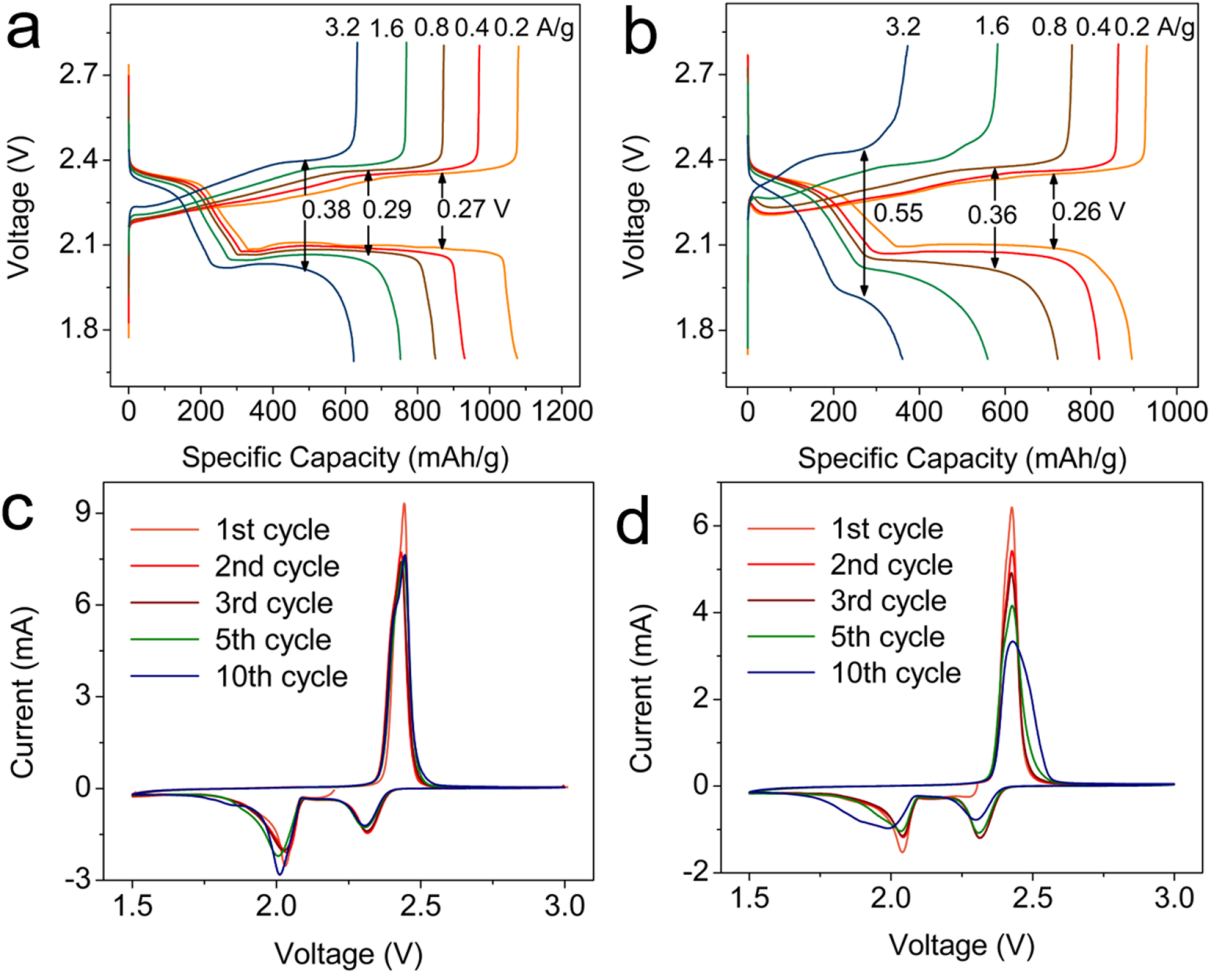
Galvanostatic charge-discharge curves of the (**a**) S-Co_3_O_4_@MCR and (**b**) S-MCR electrodes under varied currents from 0.2 to 3.2 A/g; CV curves of the (**c**) S-Co_3_O_4_@MCR and (**d**) S-MCR electrodes.

Electrochemical performances of S-Co_3_O_4_@MCR and S-MCR were examined in coin-cells, using lithium metal as a counter electrode by galvanostatic charge-discharge and cyclic voltammetry tests. Figure 4(a) displays the galvanostatic charge-discharge curves of S-Co_3_O_4_@MCR under varied currents from 0.2 to 3.2 A/g within the potential window between 1.7 and 2.8 V vs Li^+^/Li. The discharge specific capacity of S-Co_3_O_4_@MCR is ~1070 mAh/g under current density of 0.2 A/g. Two prominent plateaus can be observed at 2.33 V and 2.10 V. The charge specific capacity is ~1080 mAh/g under the same current density, and the dominating charge plateau is located at 2.35 V. The charge/discharge plateau difference, which is calculated using the 80% state of charge voltage and the 80% depth of discharge voltage values, reaches ~0.27 V. With the increased current density, both the discharge and charge specific capacities decrease and the charge/discharge plateau difference broadens. When the current density raises to 3.2 A/g, the discharge and charge capacities are still over 620 mAh/g and the charge/discharge plateau difference stays in a low value of 0.38 V. The low charge/discharge plateau difference demonstrates excellent control of Co_3_O_4_@MCR on the over-potential. Figure 4(b) displays the charge-discharge curves of S-MCR at various current densities from0.2 to 3.2 A/g. With the current density of 0.2 A/g, MCR shows a discharge specific capacity of ~890 mAh/g and charge specific capacity of ~930 mAh/g. And the charge/discharge plateau difference (~0.26 V) is at the same condition as S-Co_3_O_4_@MCR. discharge plateaus are not as prominent as that of S-Co_3_O_4_@MCR. With the increased current density, both the discharge and charge specific capacities decreased. It is noted that the polarization of MCR is much higher than that of S-Co_3_O_4_@MCR under the same current density. With the current density of 0.8 A/g, the charge/discharge plateau difference reaches 0.36 V, and with 3.2 A/g current, this difference reaches 0.55 V, which is much higher than that of S-Co_3_O_4_@MCR. What’s more, the discharge and charge plateaus tend to be inconspicuous when the current raises up to 3.2 A/g, demonstrating that Co_3_O_4_@MCR possesses the enhanced catalytic activity for the discharge/charge processes in lithium-sulfur battery.

Figure 4(c) demonstrates the CV of S-Co_3_O_4_@MCR electrode in lithium-sulfur battery. The two cathodic peaks at around 2.3 V and 2.0 V are attributed to the reduction of sulfur to high-order lithium polysulfide and eventually to lithium sulfide^46,47^. The anodic peak at around 2.4 V corresponds to the reverse conversion from Li_2_S_4_/Li_2_S to sulfur. The CV curves of S-MCR demonstrated in Fig. 4(d) convey slightly different information. The two cathodic peaks at 2.3 and 2.0 V are attributed to the reduction as stated in S-Co_3_O_4_@MCR sample. However, these peaks are shrinking during the subsequent scans. And the location for anodic peak of S-MCR which corresponds to the reverse conversion from Li_2_S_4_/Li_2_S to sulfur slightly shifts. These comparative results demonstrate that Co_3_O_4_@MCR can enhance the stability in lithium-sulfur battery.

For comparison, the electrochemical performance of bare Co_3_O_4_@MCR and MCR was conducted without sulfur under the same testing condition. Figure S4(a,b) are the galvanostatic charge-discharge curves for Co_3_O_4_@MCR and MCR under current density of 0.2 A/g. From the second galvanostatic charge-discharge cycle, no identical plateau is demonstrated for both samples and the discharge specific capacity values are only 65 and 40 mAh/g for Co_3_O_4_@MCR and MCR. This result implies that the contribution form Co_3_O_4_@MCR or MCR composites is negligible. Figure S5(a,b) are the CV curves for Co_3_O_4_@MCR and MCR composites. At the first cycle, no identical oxidation or reduction peak is displayed for both samples, which is consistent with the result in Fig. S2.

**Figure 5:**
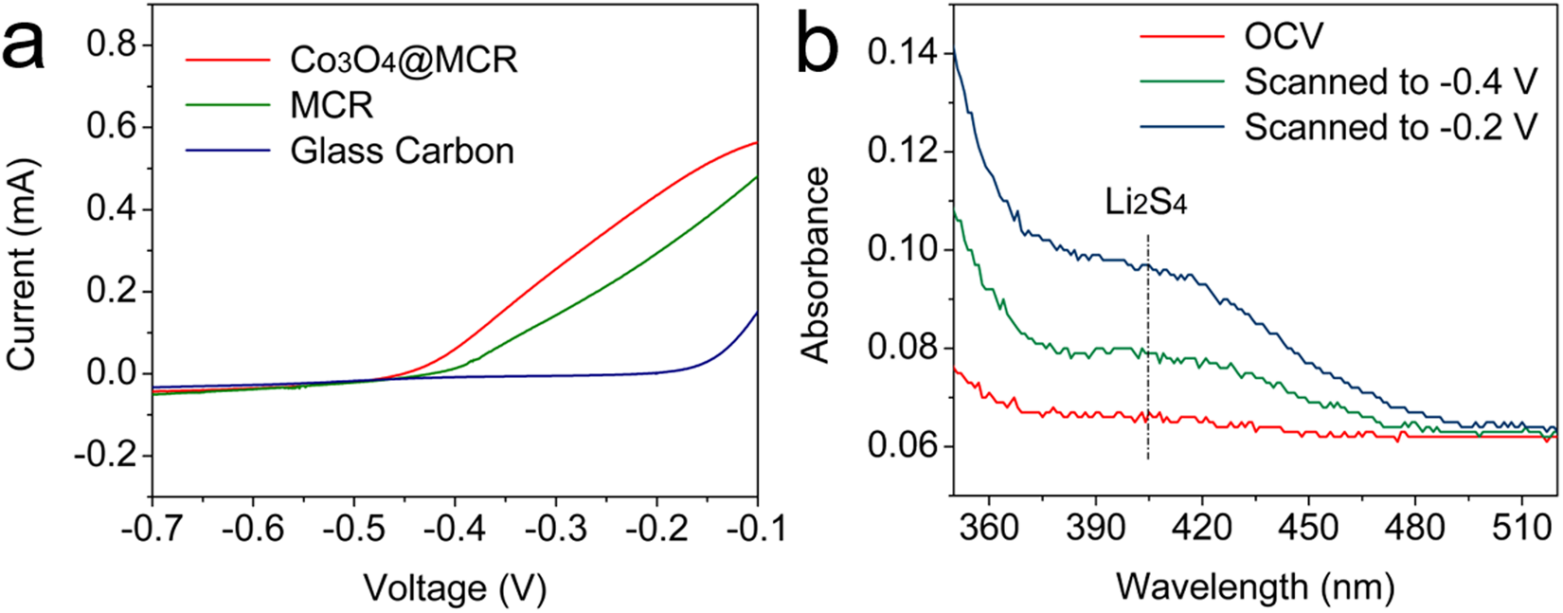
(**a**) LSV curves of sulfide oxidation reaction for Co_3_O_4_@MCR, MCR and bare glass carbon; (**b**) UV-vis adsorption spectra for the evolution measurement with Co_3_O_4_@MCR as working electrode.

To investigate the catalytic activity of the Co_3_O_4_@MCR composite, sulfide oxidation study was conducted by three electrodes electrochemical measurement for Co_3_O_4_@MCR, MCR and bare glass carbon electrodes, and the results were demonstrated in Fig. 5(a). 0.1 M Li_2_S in methanol was used as electrolyte. The charge process of lithium-sulfur battery involves sulfide oxidation reaction. Figure 5(a) demonstrates that Co_3_O_4_@MCR outperforms MCR catalyst for the sulfide oxidation reaction. Compared with the bare glass carbon electrode, Co_3_O_4_@MCR leads to improve the onset value to −0.46 V and MCR composite to −0.41 V. Co_3_O_4_@MCR composite exhibits considerably lower voltage. In galvanostatic test, either Co_3_O_4_@MCR or MCR is pasted on the surface of aluminum foil. To evaluate the influence from aluminum foil on sulfide oxidation, bare aluminum foil is used as testing electrode. As shown in Fig. S6, aluminum foil is catalytically inactive in sulfide oxidation. UV-vis adsorption spectra were applied to supply evidence during the sulfide oxidation process. Figure 5(b) exhibits the UV-vis adsorption spectra of the electrolyte with Co_3_O_4_@MCR as working electrode. There are Li_2_S_4_ signals detected at the wavelength of ~400 nm on the UV-vis adsorption spectra over −0.4 V, which demonstrated that over the onset potential Li_2_S in the electrolyte is transferred into Li_2_S_4_. In addition, the signal around 400 nm was confirmed to Li_2_S_4_ by introducing sulfur into Li_2_S solution to receive Li_2_S_4_ as reported^48^ and the corresponding evidence is exhibited in Fig. S7. In addition, the galvanostatic charge profiles for Li_2_S/CNTs, Li_2_S/CNTs/Co_3_O_4_@MCR and Li_2_S/CNTs/MCR electrodes were recorded as demonstrated in Fig. S8. The relative lower charge potential for Li_2_S/CNTs/Co_3_O_4_@MCR electrode stating the enhanced catalytic activity of Co_3_O_4_@MCR for sulfide oxidation reaction.

**Figure 6:**
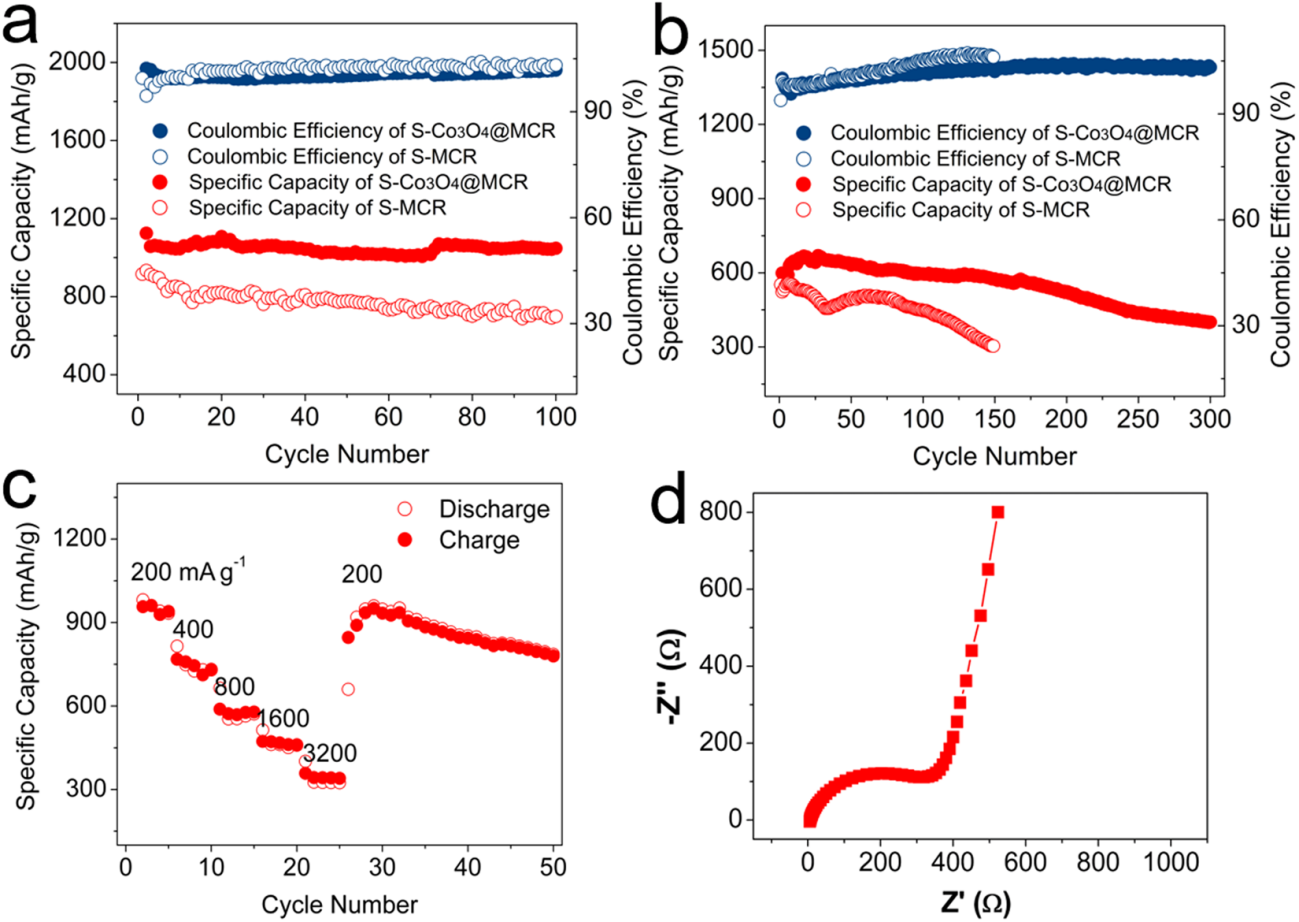
Galvanostatic cycling performance of the S-Co_3_O_4_@MCR and S-MCR electrode under (**a**) 0.2 A/g and (**b**) 3.2 A/g; (**c**) rate performance of the S-Co_3_O_4_@MCR electrode under varied currents from 0.2 to 3.2 A/g and (**d**) electrochemical impedance spectroscopy for the S-Co_3_O_4_@MCR electrode before cycling test.

Electrochemical performance of S-Co_3_O_4_@MCR in lithium-sulfur battery is demonstrated in Fig. 6. Cycling stability test of the S-Co_3_O_4_@MCR was conducted under current density of 0.2 A/g as shown in Fig. 6(a). S-Co_3_O_4_@MCR cathode exhibits excellent charge-discharge specific capacities, and the coulombic efficiency is close to 100% during the cycling. The specific capacity is around 1050 mAh/g and is stable during the cycling. Further, electrode with high S loading (5.2 mg/cm^2^) was tested under current density of 0.1 A/g with the result demonstrated as Fig. S9. With the loading increasing, the specific capacity drops to ~800 mAh/g and the capacity retention decreases during cycling. For comparison, S-MCR cathode was also tested under current density of 0.2 A/g. The charge-discharge specific capacities are lower than these of S-Co_3_O_4_@MCR cathode, and the coulombic efficiency is a little higher. The specific capacity is 930 mAh/g for the first 2 cycles and it is slowly dropping during the cycling. Both batteries were also tested under high current density of 3.2 A/g, as exhibited in Fig. 6(b). Despite the specific capacity reducing cycle after cycle, S-Co_3_O_4_@MCR demonstrates high capacity value (~410 mAh/g) over 300 cycles. But for S-MCR cathode, the performance is not satisfiable. The capacity value drops quickly within 150 cycles. Rate capability test is followed next as demonstrated in Fig. 6(c). The average charge capacities at current densities of 0.2, 0.4, 0.8, 1.6 and 3.2 A/g for the composite are ~1070, 940, 840, 750 and 600 mAh/g, respectively. After the current rate resettled to 0.2 A/g, the capacities return to an elevated level. This reflects high reaction kinetics of S-Co_3_O_4_@MCR during battery reaction progresses. Electrochemical impedance spectroscopy is performed in the range from 1.0 MHz to 0.1 Hz at open circuit potential before cycling test for the S-Co_3_O_4_@MCR cathode. The resultant Nyquist plots are presented in Fig. 6(d). The high frequency semi-circle mainly refers to the charge transfer resistance (Rct) and the low-frequency line region refers to Warburg diffusion process (Zw). The Rct value is calculated to be 317 Ω for S-Co_3_O_4_@MCR cathode, exhibiting significantly electronic conductivity which is beneficial from crystal carbon substrate matrix and is appreciative for electrode materials.

## Conclusions

Co_3_O_4_ nano-particle embedded mesoporous carbon rod was prepared through a template method to accommodate sulfur as cathode for lithium-sulfur battery. The Co_3_O_4_@MCR composite mixed with sulfur demonstrates excellent electrochemical performance and enhanced catalytic performance in lithium-sulfur battery. Under current density of 0.2 A/g, a specific discharge capacity above 1000 mAh/g can be achieved. The Co_3_O_4_@MCR composite reduces the over-potential during charge process due to the electro catalytic behavior. Besides, sulfide oxidation measurement was conducted, and the data obtained is helpful in evaluating the performance of the electrode for sulfide oxidation in lithium-sulfur batteries.

## Electronic supplementary material

Supplementary Materials
