# Supplementary material for: Co3O4-NP embedded mesoporous carbon rod with enhanced electrocatalytic conversion in lithium-sulfur battery
Source: Sci Rep. 2018 Oct 31;8:16133. doi: 10.1038/s41598-018-34195-z (PMC6208390; doi:10.1038/s41598-018-34195-z)
Supplement: Supplementary file 1 — Supplementary Materials [file 41598_2018_34195_MOESM1_ESM.docx]

**Co_3_O_4_-NP embedded mesoporous carbon rod with enhanced electrocatalytic conversion in lithium-sulfur battery**

Shaofeng Wang, ^a^ Xianhua Hou, ^a^* Zeming Zhong, ^a^ Kaixiang Shen, ^a^ Guangzu Zhang, ^b^ Lingmin Yao ^c^ and Fuming Chen ^a^*

a. Guangdong Engineering Technology Research Center of Efficient Green Energy and Environment Protection Materials, Guangdong Provincial Key Laboratory of Quantum Engineering and Quantum Materials, School of Physics and Telecommunication Engineering, South China Normal University, Guangzhou 510006, PR China.

b. School of Optical and Electronic Information, Huazhong University of Science and Technology, Wuhan, 430074, PR China.

c. School of Physics and Electronic Engineering, Guangzhou University, Guangzhou, 510006, PR China.

**Characterization**

The information about the pore size and distribution of Co_3_O_4_@MCR and MCR was investigated by N_2_ adsorption/desorption isotherms, and the pore size distribution result based on Barret-Joyner-Halenda method is shown in **Figure-S1**.





**Figure-S1** BJH pore distribution curves of the as-prepared Co_3_O_4_@MCR and MCR samples

To offer evidence for the accommodation capability of Co_3_O_4_@MCR, the EDX elemental mapping images for S/Co_3_O_4_@MCR were taken as the result shown in Figure-S2. The S mapping signal is spreading though the whole sample district, suggesting that S is well accommodated inside the Co_3_O_4_@MCR composite.

**
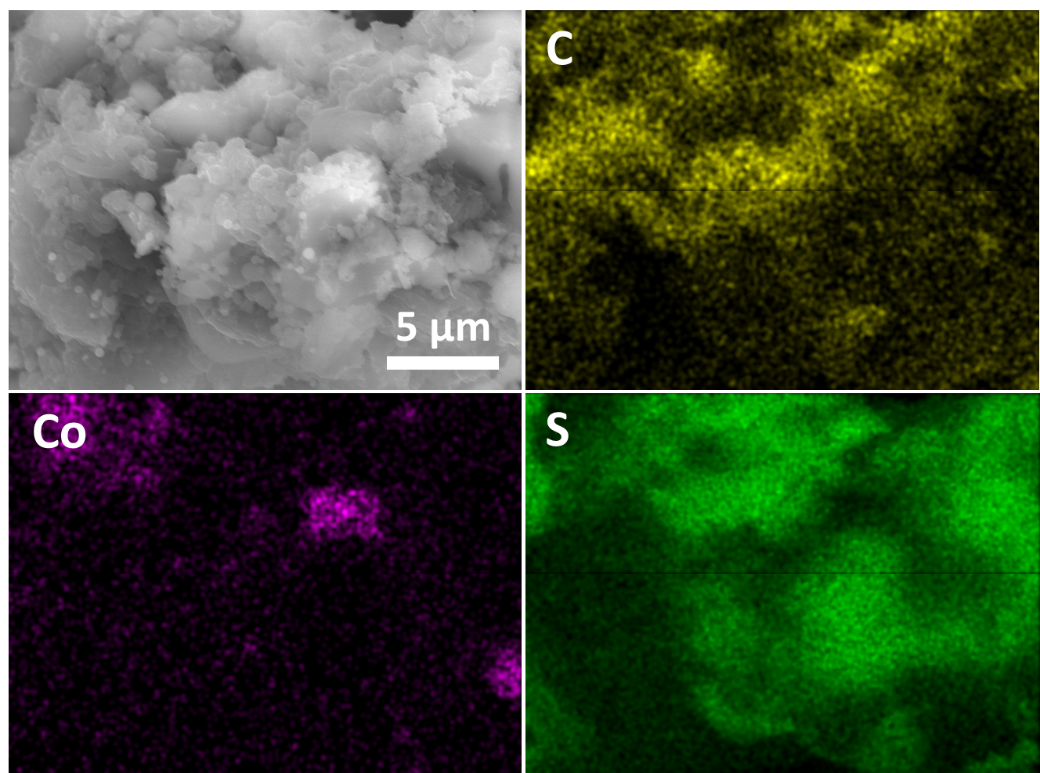
**

**Figure-S2** EDX elemental mapping images of carbon, cobalt and sulfur signals for the as-prepared Co_3_O_4_@MCR sample

**
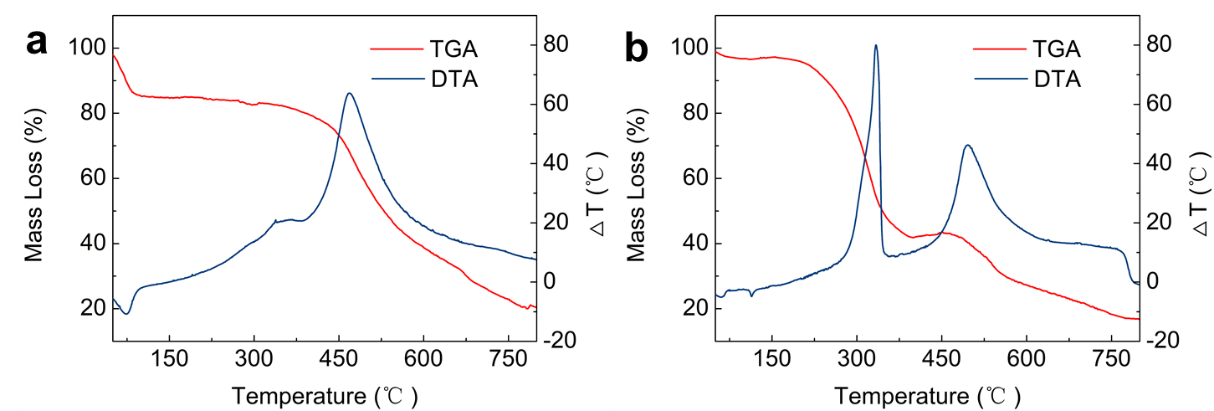
**

**Figure-S3** Thermogravimetric analysis of the (a) Co_3_O_4_@MCR and (b) S-Co_3_O_4_@MCR

**Electro-chemical measurement**

Figure-S3 (a) & (b) are the galvanostatic charge-discharge curves for Co_3_O_4_@MCR and MCR electrodes under current density of 200 mA g^-1^, respectively. For the original cycle, the discharge specific capacity values are respective 140 and 100 mAh g^-1^ for Co_3_O_4_@MCR and MCR electrodes between 1.2 and 2.8 V vs. Li^+^/Li. For the second galvanostatic charge-discharge cycle, the discharge specific capacity values are respective 65 and 40 mAh g^-1^ for Co_3_O_4_@MCR and MCR electrodes. This result implies that both Co_3_O_4_@MCR and MCR composites contribute very little specific capacity.


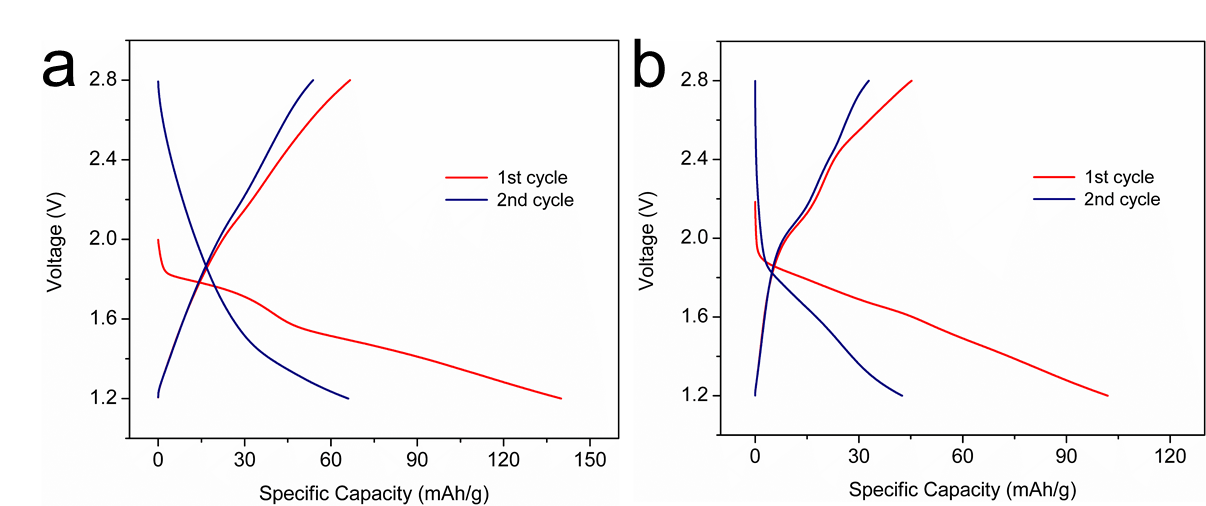


**Figure-S4** Galvanostatic charge-discharge curves of the (a) Co_3_O_4_@MCR and (b) MCR electrodes under varied currents from 200 to 3200 mA g^-1^


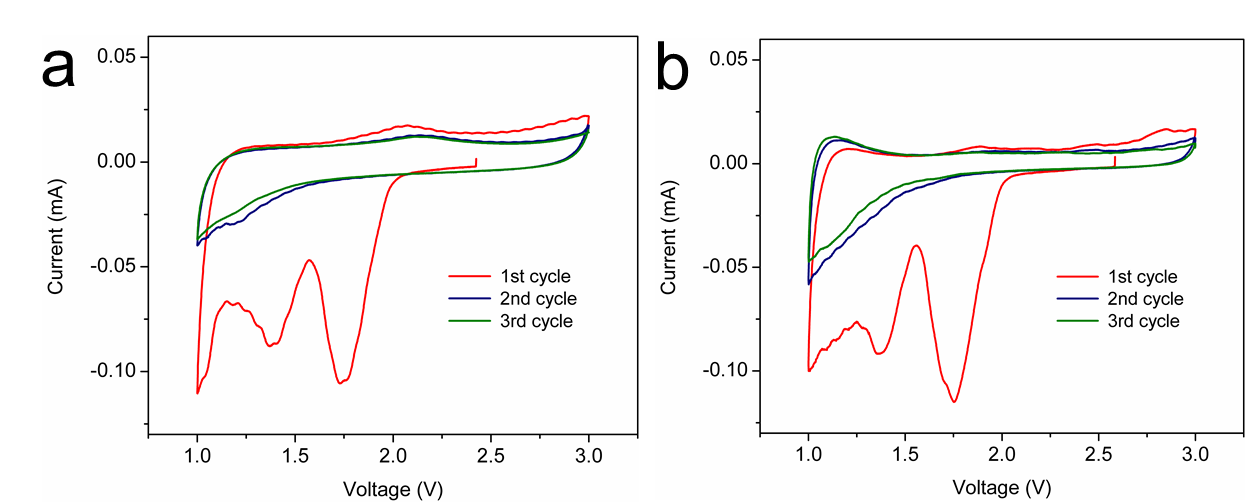


**Figure-S5** CV curves of the (c) Co_3_O_4_@MCR and (d) MCR electrodes





**Figure-S6** LSV curves of sulfide ion evolution reaction for Al foil

**UV-vis adsorption characterization**

Li_2_S_4_ was received by adding sulfur into Li_2_S solution. To locate the UV-vis adsorption peak for Li_2_S_4_ in spectra, growing amount of S was added into Li_2_S solution. As the amount of S added into the Li_2_S solution is elevated, the signal around 400 nm on UV-vis adsorption spectra becomes prominent. That the signal around 400 nm on UV-vis adsorption spectra was confirmed to Li_2_S_4_, as the evidence exhibited in Figure-S6.


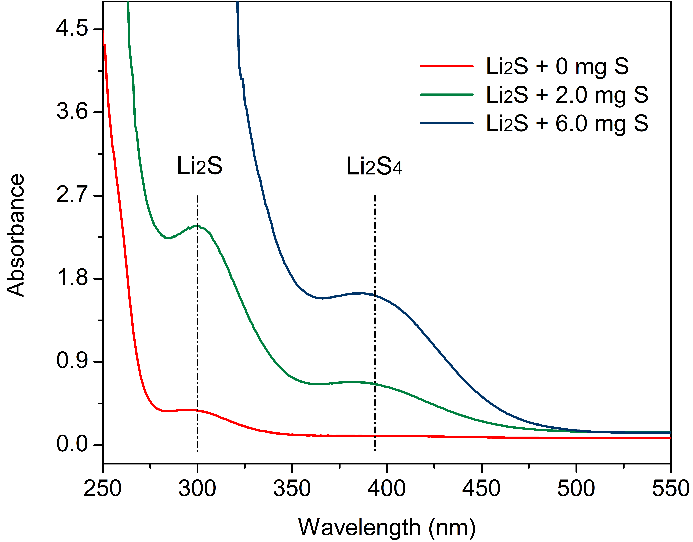


**Figure-S7** UV-vis adsorption spectra for Li_2_S and Li_2_S_4_ in methanol


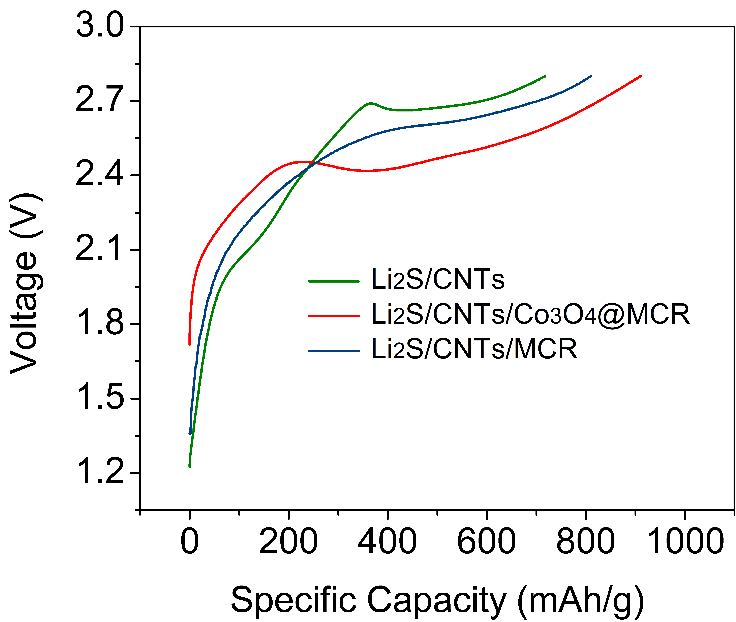


**Figure-S8** Galvanostatic charge profiles for Li_2_S/CNTs, Li_2_S/CNTs/Co_3_O_4_@MCR and Li_2_S/CNTs/MCR electrodes

Li_2_S/CNTs was received by heating Li_2_SO_4_ with CNTs in N_2_ atmosphere under 800 ℃. To form Li_2_S/CNTs/Co_3_O_4_@MCR and Li_2_S/CNTs/MCR electrodes, 80 wt.% of Li_2_S/CNTs, 10 wt.% catalyst and 10 wt.% pvdf were mixed to slurry and coated on Al foil.


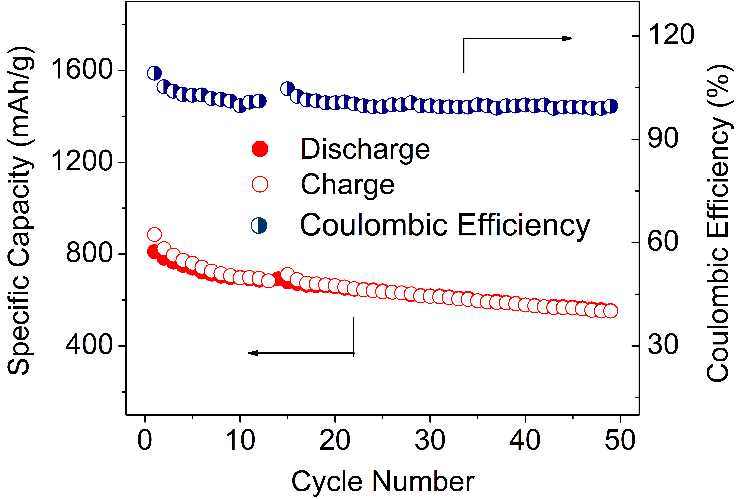


**Figure-S9** Galvanostatic cycling performance of the S-Co_3_O_4_@MCR electrode with S loading of 5.2 mg/cm^2^ under current density of 0.2 A/g
